# Antimicrobial EU@Ag-MOF/PLA composite films enhance postharvest quality of strawberries by mitigating oxidative stress and modulating microbial communities

**DOI:** 10.1016/j.fochx.2025.103377

**Published:** 2025-12-04

**Authors:** Yuyue Qin, Yurou Wang, Yiwei Huang, Haiyan Chen, Yongliang Zhuang, Qiuming Liu, Thanapop Soteyome, Bifen Zhu, Charles Brennan

**Affiliations:** a Yunnan International Joint Laboratory of Green Food Processing, Faculty of Food Science and Engineering, Kunming University of Science and Technology, Kunming 650550, China; b Rajamangala University of Technology Phra Nakhon, Bangkok 10300, Thailand; c Department of Food Science and Engineering, School of Agriculture and Biology, Shanghai Jiao Tong University, Shanghai 200240, China; d School of Science, Royal Melbourne Institute of Technology University, Melbourne 3000, Australia

**Keywords:** Polylactic acid, Silver-based metal-organic framework, Eugenol, Antibacterial packaging, Strawberry

## Abstract

Strawberries are highly perishable fruits susceptible to rapid postharvest deterioration. This study investigated the efficacy of EU@Ag-MOF/PLA composite films for postharvest strawberry preservation. The 3EU@Ag-MOF/PLA formulation demonstrated optimal performance in maintaining physicochemical properties, reducing reactive oxygen species accumulation, and enhancing antioxidant enzyme activities. Notably, this composite film suppressed hydrogen peroxide, superoxide anion, and malondialdehyde content by 28.44 %, 37.33 %, and 29.91 %, respectively, compared to control packaging after 10 days. Pathogen challenge studies with Botrytis cinerea and Rhizopus stolonifer revealed that this composite effectively balances reactive oxygen species production and scavenging mechanisms during storage. High-throughput sequencing showed that 3EU@Ag-MOF/PLA packaging preserved more balanced microbial community structures and dramatically reduced the relative abundance of pathogenic fungi by day 10. These findings demonstrate that 3EU@Ag-MOF/PLA composite film represent a promising active packaging solution for extending the shelf life of highly perishable fruits by simultaneously addressing fungal proliferation, oxidative stress, and microbial ecology

## Highlights

• Active packaging enhanced the antioxidant enzyme activities of POD, CAT, and SOD in strawberries.
• Active film reduced the reactive oxygen species-related H_2_O_2_, O2^•−^ and MDA in strawberries.
• Active film significantly reduced the fungal and bacterial Alpha diversity in strawberries.
• Active film can delay the deterioration of strawberries and maintain the quality of strawberries.

## 1 Introduction

Strawberries are an excellent source of antioxidants and total phenolic compounds, and are highly regarded for their rich nutrients and unique flavor (Tahir, Fatima, Fatima, & Ali, 2024). However, their extreme susceptibility to mechanical damage (Xu et al., 2025), microbial infection, and physiological metabolic imbalances leads to shortened shelf life and reduced commercial value (Wang et al., 2024). Postharvest quality deterioration primarily manifests as fruit shrinkage due to moisture loss, decay caused by microorganisms (such as *Botrytis cinerea* and *Rhizopus stolonifer*) (Wang et al., 2024; Xu et al., 2025). Additionally, the thin wax layer on strawberry surfaces facilitates pathogen spore adhesion and germination, with fungal proliferation rates increasing 3–5 folds in environments exceeding 90 % humidity (Al-Tayyar, Youssef, & Al-Hindi, 2020). Conventional approaches to controlling these pathogens have primarily relied on synthetic fungicides; however, increasing consumer concerns regarding chemical residues, pathogen resistance development, and environmental impacts have necessitated the exploration of alternative preservation strategies (Javed et al., 2024; Karnwal et al., 2025).

In recent years, active packaging technologies have emerged as promising alternatives to direct chemical applications for preserving fresh produce. Active packaging refers to systems that actively interact with the packaged product or its environment to extend shelf life and maintain quality. Among various active packaging approaches, antimicrobial packaging has shown particular promise for fresh fruits by creating a protective microenvironment that suppresses pathogen proliferation without direct contact between antimicrobial agents and fruit surfaces (Zhang et al., 2023).

Within antimicrobial materials, Metal-organic frameworks (MOFs)-derived materials exhibit remarkable germicidal activity through controlled ion release (Qian, Zhang, Li, Shuai, & Wang, 2021). Ag-MOF not only inherits the excellent adsorption capacity, drug-loading capability, and structural stability from MOFs, but also gains antimicrobial properties against a broad spectrum of microorganisms due to the incorporated silver nanoparticles (Li et al., 2023), or generating reactive oxygen species (ROS) to inhibit the growth of bacteria and molds (Elmehrath et al., 2024). By *in situ* growth, γ-cyclodextrin metal-organic frameworks (γ-CD-MOFs) were integrated onto the chitosan-cellulose film. The encapsulation and sustained-release of carvacrol were achieved through γ-CD-MOFs. The inhibition rate of γ-CD-MOFs film against *Escherichia coli*, *Staphylococcus aureus* and *Botrytis cinerea* reached more than 99 %. Treated with this film, the shelf life of strawberries was extended to 7 days (Min et al., 2024). In our preliminary research, we utilized polylactic acid (PLA) as a film-forming matrix to incorporate the natural antibacterial agent eugenol (EU) into Ag-MOF. The composite film exhibits no cytotoxicity while demonstrating outstanding antioxidant and antibacterial performance. Notably, this composite film reduces the release rate of EU, thereby offering a long-lasting and safe slow-release technology for postharvest preservation of strawberries (Qin et al., 2024).

This study investigated the preservation effect of silver-loaded metal-organic framework/polylactic acid (Ag-MOF/PLA) at different concentrations (1 %, 3 %, and 5 %) composite films for the quality of strawberries under refrigeration conditions. By comprehensively evaluating physicochemical properties, oxidative stress parameters, enzyme activities, and microbial community dynamics, this research aims to elucidate the multifaceted mechanisms by which Ag-MOF/PLA composite films influence strawberry preservation. Additionally, challenge experiments with *Botrytis cinerea* and *Rhizopus stolonifera* provide insights into the efficacy of these composite films under conditions of elevated disease pressure, simulating real-world postharvest scenarios. This comprehensive investigation advances our fundamental understanding of the complex interactions between active packaging materials, fruit physiology, and microbial ecology in postharvest systems. The findings will contribute to the development of more effective, sustainable preservation strategies for highly perishable fruits, addressing the critical global challenges of food loss reduction and food security enhancement.

## 2 Material and methods

### 2.1 Materials

Strawberries were purchased from Run Green Farm, Kunming, China. Gallic acid, Folin-Ciocalteau reagent, 2,6-dichloroindophenol solution, ascorbic acid and oxalic acid were purchased from Sinopharm Chemical Reagent Co. Ltd. (Shanghai, China), and the H_2_O_2_, •O_2_-, MDA, POD, SOD, CAT and PPO kits were purchased from Nanjing Jianjian Bio-Technology Co. All the chemical reagents were of analytical grade.

### 2.2 Preparation of films and preservation of strawberries

The preparation of Ag@MOF nano particle and Ag@MOF/PLA films were based on our previous study. The films were prepared by solution casting method, 1 % Ag@MOF (wt), 3 % Ag@MOF (wt), 5 % Ag@MOF (wt) and 2 g PLA powder, 10 % acetyltributyl citrate plasticizer (ATBC) (wt) were mixed to make PLA nano-antimicrobial slow-release composite films and named as 1EU@Ag-MOF/PLA film, 3EU@Ag-MOF/PLA film, and 5EU@Ag-MOF/PLA film, respectively (Qin et al., 2024).

Fresh strawberries with a maturity level of 70–80 % free from pests and mechanical damage were selected and placed in plastic boxes and sealed by PLA film, 1EU@Ag-MOF/PLA film, 3EU@Ag-MOF/PLA film, and 5EU@Ag-MOF/PLA film, respectively. The weight of a single strawberry was approximately 18 g. Each plastic boxes contained 15 strawberries, and 18 boxes of each group. Plastic boxes were disinfected with 75 % alcohol and subjected to ultraviolet irradiation for 30 min. The strawberries were stored in a refrigerator at 4 °C for 10 days. Samples were taken every two days, and the changes in various indicators were measured. Measurements were repeated three times (Nouman, 2025).

### 2.3 Measurement postharvest quality of strawberries

#### 2.3.1 Appearance and color

Strawberry appearance changes during storage were recorded by taking photos at regular intervals, and color changes were determined using a colorimeter by measuring *L* (brightness), *a* (red-green value) of strawberries (Qin et al., 2025).

#### 2.3.2 Weight loss, firmness and total soluble solid

Weight loss of 15 strawberries was calculated according to the following equation:

(1) $$\mathrm{Weight loss}=\frac{m_{0}-m_{t}}{m_{0}}$$

Where, m_0_ denotes the initial mass of strawberries, m_t_ denotes the weight of strawberries on each sampling day.

The firmness of the strawberries was measured by texture meter with a 2 mm diameter needle-type probe (P/2), and the probe speed was set to 2 mm/s. The total soluble solid (TSS) of each group of strawberries was determined by using a refractometer (Qin et al., 2025).

#### 2.3.3 Electrolyte permeability and respiration rate

Strawberries (5 g) were chopped and placed in a test tube containing 15 mL of distilled water shocked and shaken well, using a conductivity meter to determine the initial conductivity P_0_ of strawberries. The sample was boiled for 10 min, then cooled to room temperature. The conductivity P_1_ was determined. Each set of samples was analyzed in triplicate. The formula for calculating electrolyte permeability is as follows:

(2) $$\mathrm{Electrolyte}\;le\mathrm{akage}\left(\%\right)=\frac{P_{0}}{P_{1}}\times 100$$

Strawberries were placed in the respiration chamber of the fruit and vegetable respirometer with a time interval of 900 s to determine the strawberry respiration rate during storage (Iqbal et al., 2024).

#### 2.3.4 Total phenols and ascorbic acid in strawberries

Strawberries (5 g) were mixed with 20 mL of pre-cooled 70 % ethanol and ground in an ice bath. Then, it was centrifuged at 4000 rpm for 15 min, and the supernatant was collected as the strawberry extract. Strawberry extract (0.5 mL) was placed in a 50 mL centrifuge tubes, 25 mL of 10 % Folin-ciocalteu reagent was added. Na_2_CO_3_ (7.5 %, 2 mL) solution was added, and the reaction was shaking for 60 min in a dark place at room temperature. The absorbance value of the solution was measured at 765 nm (Ummer et al., 2024; Zhang et al., 2021). Gallic acid was used as the standard. The total phenols (TPC) were calculated by following equation:

(3) $$\mathrm{TPC}\left(\mathrm{mg}\;\mathrm{GAE}/g\right)=\frac{C\times W\times V}{m\times 1000}$$

Where C is the concentration obtained from the standard curve, μg/mL; W is the dilution of the extract; V is the total volume of strawberry extract, mL; m is the mass of the strawberry sample, g.

Strawberries (10 g) and oxalic acid solution (20 g/L) were quickly crushed into a homogeneous paste in the dark. The strawberry paste (5 g) was placed in a volumetric flask, and the oxalic acid solution was added to reach 50 mL. Then, white clay (2 g) was added to decolorize the solution, and the mixture was centrifuged at 4000 rpm for 10 min. The supernatant was taken as the titration sample. The accurate concentration of the 2,6-dichloroindophenol solution is 0.231 mg/mL, and it was titrated using a standard vitamin C solution. The titration sample (10 mL) was titrated with the calibrated 2,6-dichloroindophenol solution, and the volume of consumption was recorded (Zhang et al., 2021). The ascorbic acid content in strawberries was determined by the following formula:

(4) $$\mathrm{AsA}\;\mathrm{content}\left(\frac{\mathrm{mg}}{100g}\right)=\frac{\left(V-V_{0}\right)\times T\times A}{m}\times 100$$

Where V represents the volume of 2,6-dichloroindophenol solution used to titrate the specimen, mL; V_0_ is the volume of 2,6-dichloroindophenol solution consumed by the blank, mL; T is the titration degree of 2,6-dichloroindophenol solution, mg/mL; A is the dilution multiplier; m is the mass of specimen, g.

#### 2.3.5 Determination of oxidative stress markers and antioxidant enzyme activities

Hydrogen peroxide (H_2_O_2_), superoxide anion radical (•O_2_-) and malondialdehyde (MDA) in strawberry during storage were determined by using commercial assay kit produced by Nanjing Jiancheng Biotechnology Co., LTD. (Nanjing, China). The number of the kits were A064–1-1, A052–1-1 and A003–1-2, respectively.

Peroxidase activity (POD), catalase activity (CAT), superoxide dismutase activity (SOD) and polyphenol oxidase activity (PPO) in strawberry were determined using the kit produced by Nanjing Jiancheng Biotechnology Co., LTD. (Nanjing, China). The number of the kits were A084–3-1, A007–1-1, A001–1-2 and A136–1-1, respectively.

### 2.4 Preservation of strawberries infected by *Botrytis cinerea* and *Rhizopus stolonifera*

Based on the results from Section 2.3, among the five films, the best post-harvest preservation effect for strawberries was 3EU@Ag-MOF/PLA film. Therefore, in this section, 3EU@Ag-MOF/PLA film was selected as the packaging material, and the control group was PLA film. The strawberries were inoculated with *Botrytis cinerea* (*B. cinerea*) and *Rhizopus stolonifera* (*R. stolonifera*). Changes in the antioxidant enzyme systems (including POD, SOD, CAT, and PPO activities), ROS products (•O_2_- and H₂O₂), and MDA content of the infected strawberries were monitored. Additionally, combined with the biological genomics 16S and ITS sequencing techniques, the impact of the 3EU@Ag-MOF/PLA film on the biodiversity of the infected strawberries was investigated.

#### 2.4.1 Preparation of *Botrytis cinerea* and *Rhizopus stolonifera* suspensions

The refrigerated *B. cinerea* and *R. stolonifera* were thawed at room temperature for 30 min, then the two pathogenic fungi were inoculated into PDA medium and cultured in an incubator at 27 °C for 7–10 days. The mycelium was rinsed with sterile phosphate buffer and then transferred into a sterile centrifuge tube. It was centrifuged at 4000 rpm for 10 min, and the precipitate was collected. The spore concentration of each fungal suspension was adjusted to 10^5^–10^6^ CFU/mL by using a hemocytometer (Qin et al., 2025).

#### 2.4.2 Inoculated strawberry treatment

Fresh strawberries without mechanical damage were rinsed three times with sterile water and dried naturally. The bacterial suspension was evenly sprayed on the strawberries with a sterile spray bottle. After the surface of the strawberries was dry, the strawberries were randomly placed in a plastic box that had been sterilized with 75 % alcohol and irradiated with UV light for 30 min. The box was sealed with a PLA film and a 3EU@Ag-MOF/PLA film, which had also been irradiated with UV light for 30 min. At the same time, the surface of the strawberries was sprayed with an equal amount of sterile saline, then placed in the plastic box and sealed with the PLA film and 3EU@Ag-MOF/PLA film. The group inoculated with *R. stolonifera* was named FR, the group inoculated with *B. cinerea* was named FB, and the control group was named C. The PLA group was numbered as 1, and the 3EU@Ag-MOF/PLA group was numbered as 2. The specific names were shown in Table 1. Each plastic boxes contained 15 strawberries, and 18 boxes of each group. Finally, strawberries of all the groups were placed in refrigeration for 10 days at 4 °C and 76 % relative humidity, and samples were taken on days 0, 2, 4, 6, 8, and 10 in the ultra-clean bench to determine the corresponding indexes, respectively.

**Table 1:** Experimental apparatus.

	PLA fim	3EU@Ag-MOF/PLA film
saline solution(C)	C_1_	C_2_
*Rhizopus stolonifera* (FR)	F_1_R	F_2_R
*Botrytis cinerea*s (FB)	F_1_B	F_2_B

#### 2.4.3 Determination of oxidative stress markers and antioxidant enzyme activities

The methods for determining the contents of H_2_O_2_, •O_2_- and MDA content in the inoculated strawberries were the same as described in 2.3.5.

The antioxidant enzyme systems of inoculated strawberries were determined including POD, SOD, CAT and PPO, and the determination of these enzyme activities were the same as described in 2.3.5.

#### 2.4.4 Amplicon high-throughput sequencing and analytical methods

Total genomic DNA was extracted using the CTAB method, and its concentration and purity were assessed by electrophoresis on a 1 % agarose gel. The 16SV4 region primers 341F (5’-CCTAYGGGRBGCASCAG-3′) and 806R (5’-GGACTACNNGGGGTATCTAAT-3′) were used to amplify the 16SV3-V4 rRNA genes to detect bacterial diversity. Meanwhile, fungal diversity was detected by using primers ITS5-1737F (5’-GGAAGTAAAAGTCGTAACAAGG-3′) and ITS2-2043R (5′- GCTGCGTTCTTCATCGATGC-3′) amplification of the ITS gene in the ITS1 region was performed. All PCR reactions were performed using 15μLPhusion® High-Fidelity PCR Master Mix (New England Biolabs), 2 μM forward and reverse primers, and approximately 10 ng of template DNA. Sequencing libraries were generated using the TruSeq® DNA PCR-Free Sample Preparation Kit (Illumina, USA) and index codes were added. Fluorometer (Thermo Scientific) and Agilent Bioanalyzer2100 system were used for evaluation. Finally, libraries were sequenced on the Illumina NovaSeq platform to generate 250 bp paired-end reads (Jiang et al., 2025).

### 2.5 Statistical analysis

All data were expressed as mean ± standard deviation (SD) of three independent replicates at least. The results were analyzed using SPSS 21.0 software by one-way analysis of variance (ANOVA), followed by Duncan's multiple-range test. Differences at *p* < 0.05 were considered significant. All graphs were plotted using Origin 2024.

## 3 Results and discussion

### 3.1 The influence of composite films on the postharvest preservation quality of strawberries

#### 3.1.1 The appearance and color change of strawberries

**Fig. 1:**
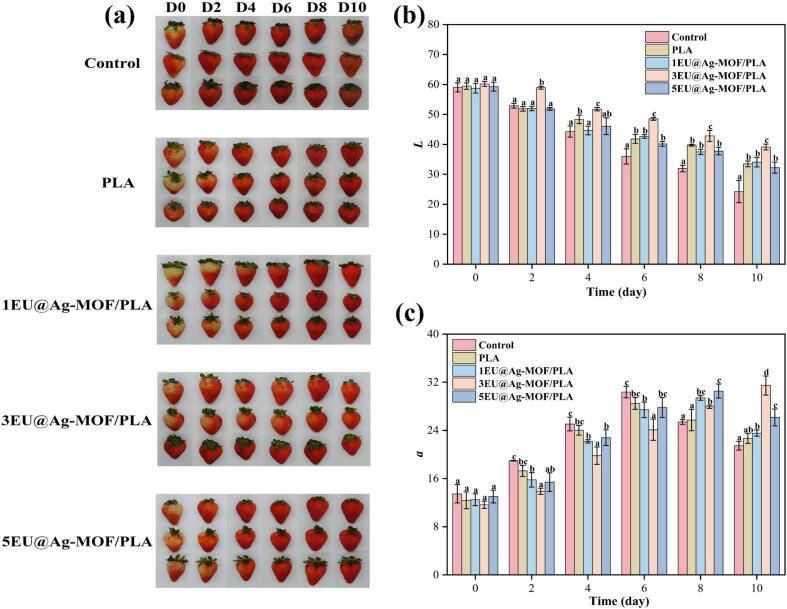
The changes in appearance (a), *L* (b) and *a* (c) during strawberry storage. Each point represents the mean value, with error bars indicating the standard error of the mean. Statistical significance between different treatments at each time point is denoted by different letters, with a significance level set at *p* < 0.05. Letter “a” represents the lowest value at each storage time.

Fig. 1a shows the visual appearance of strawberries over a 10-day storage under different packaging conditions. Strawberries in the control group and those packaged with PLA began to show obvious deterioration from the 6th day, and the surface dehydration and darkening became more severe by the 10th day. In contrast, strawberries packaged with EU@Ag-MOF/PLA composite films maintained better visual quality throughout the storage period. Remarkably, the 3 EU@Ag-MOF/PLA treated strawberries retained their structural integrity even on day 10, maintained bright red peel coloration without any mold formation.

As shows in Fig. 1b, the *L* value, representing fruit brightness, decreased progressively in all treatments, indicated darkening of strawberry surfaces. At day 0, no significant differences were observed among treatments (*p* > 0.05). By day 6, the control samples exhibited significance lower *L* values (35.99 ± 2.53) compared to EU@Ag-MOF/PLA treatments, with 3EU@Ag-MOF/PLA maintained the highest brightness (*p* < 0.05). This trend continued through day 10, where the control (24.23 ± 3.69) and PLA (33.5 ± 0.95) treatments showed substantially greater reductions in *L* values compared to EU@Ag-MOF/PLA films (*p* < 0.05). The 3EU@Ag-MOF/PLA film demonstrated superior performance in maintaining strawberry brightness throughout the storage period, suggested an optimal concentration for preservation effectiveness. The *a* value increased initially and then declined in most treatments, whereas 3EU@Ag-MOF/PLA samples exhibited a gradual increase throughout storage (Fig. 1c). These differences were attributed to the slow-release antimicrobial effect of the EU@Ag-MOF/PLA films which could effectively inhibit the growth of microorganisms and delay the metabolism of the fruits (Qin et al., 2024).

#### 3.1.2 Weight loss, firmness and TSS of strawberry

**Fig. 2:**
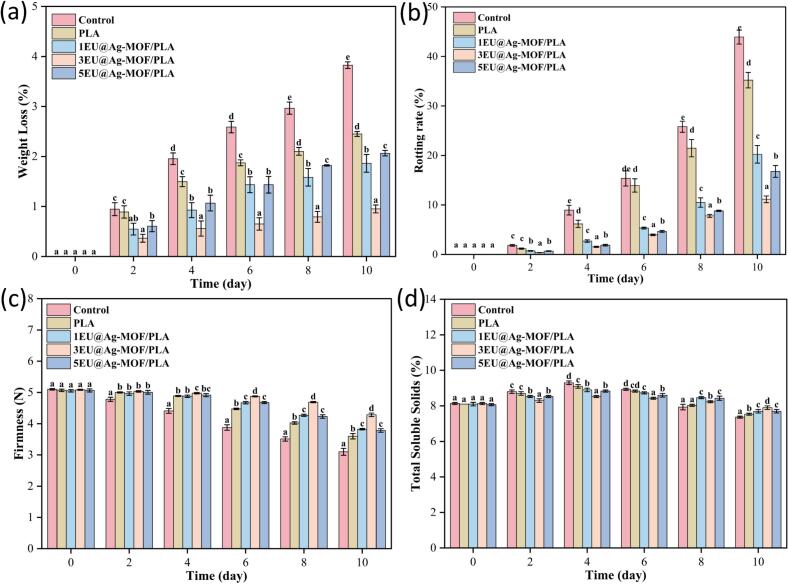
The changes in weight loss (a), rotting rate (b), firmness (c) and TSS content (c) during strawberry storage. Each point represents the mean value, with error bars indicating the standard error of the mean. Statistical significance between different treatments at each time point is denoted by different letters, with a significance level set at *p* < 0.05. Letter “a” represents the lowest value at each storage time.

The delicate skin of strawberries makes them highly susceptible to water loss during storage. As shows in Fig. 2a, the weight loss rate of strawberries in all groups gradually increased during storage time. The weight loss of strawberries in the control group was higher than other groups (*p* < 0.05). The lowest weight loss was observed in the 3EU@Ag-MOF/PLA group (*p* < 0.05). On day 10, the rotting rate of strawberries in control, PLA, 1EU@Ag-MOF/PLA, and 5EU@Ag-MOF/PLA groups were increased to 43.9 %, 35.2 %, 20.2 %, and 16.7 %, respectively. In comparison, strawberries in 3EU@Ag-MOF/PLA film had a rotting rate of 11.1 % (Fig. 2b). The 3EU@Ag-MOF/PLA film acted as a semi-permeable barrier to gas and water, thus reduced respiration and water loss and counteracted the dehydration and shrinkage of the fruits (Qin et al., 2024).

Fig. 2c shows the firmness changes of strawberries during storage. The results demonstrated that the firmness considerably decreased for all packaging. On day 10, the firmness of strawberries in PLA, 1EU@Ag-MOF/PLA, 3EU@Ag-MOF/PLA, and 5EU@Ag-MOF/PLA treatment groups were reduced by 39.2 %, 29.0 %, 24.4 %, 15.7 %, and 25.3 %, respectively. Among them, the hardness reduction in the 3EU@Ag-MOF/PLA treatment group was the smallest (*p* < 0.05). This indicated that a moderate amount of EU slowed down the degradation of pectin and cellulose, inhibited the activity of cell wall-degrading enzymes, and thereby helped maintain the normal structure of the cell wall (Tian et al., 2024).

Fig. 2d shows the changes in the total soluble solids (TSS) of strawberries. The TSS of strawberries in all groups showed an increasing and then decreasing. The slight increase in TSS in the pre-storage period may be attributed to the decomposition of the cell wall, decrease in respiration rate, and increase in dry matter because of water loss (Salaria, Patel, Sridhar, Chawla, & Sharma, 2025). Some research also suggested this may be attributed to the hydrolysis of starch and other polysaccharides into soluble sugars (Iñiguez-Moreno & González-González, 2025). Contrary to our findings, a few studies reported an increase in TSS (Iñiguez-Moreno & González-González, 2025; Pham et al., 2024). These differences might be attributed to the different diversity and maturation stages of strawberries at the beginning of the storage period.

#### 3.1.3 Electrolyte permeability and respiration rate of strawberries

**Fig. 3:**
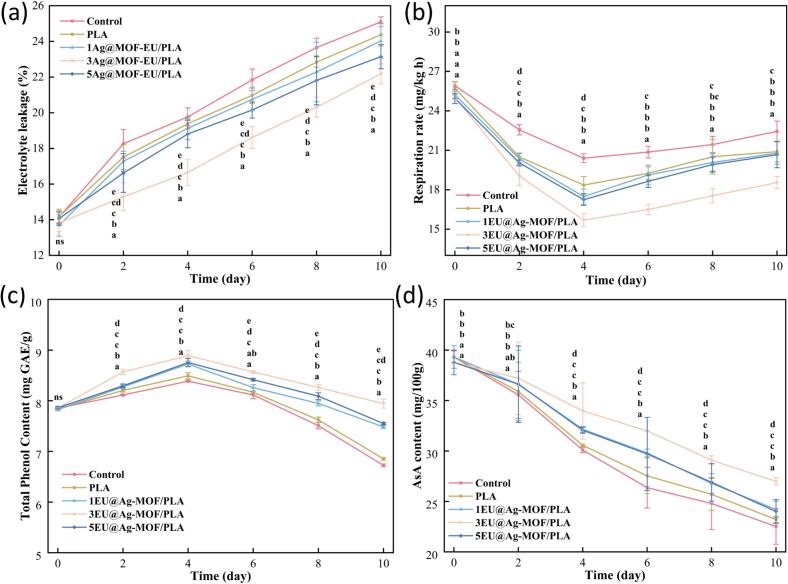
Electrolyte leakage (a), respiration rate (b), total phenol (c) and ascorbic acid (d) contents during strawberry storage. Each point represents the mean value, with error bars indicating the standard error of the mean. Statistical significance between different treatments at each time point is denoted by different letters, with a significance level set at *p* < 0.05. The arrangement of letters from top to bottom indicates the statistical ranking from highest to lowest value at each storage time. The notation “ns” indicates non-significant.

Electrolyte permeability usually reflects the degree of cell membrane integrity or damage in strawberries, and is correlated with their sensory quality, texture, appearance, and shelf life. The higher the electrolyte permeability, the greater the degree of cell membrane damage, and consequently the poorer the fruit quality (Liu et al., 2024). As shows in Fig. 3a, there was a gradual increase in the electrolyte leakage of strawberries during storage. Among them, the 3EU@Ag-MOF/PLA treatment group showed the lowest electrolyte permeability, and the control group showed the highest electrolyte permeability (*p* < 0.05). These results indicated that the 3EU@Ag-MOF/PLA film could inhibit the damage of lipid peroxidation to the cellular membrane structure of strawberries.

A higher respiration rate in strawberries indicates faster consumption of nutrients such as sugars, vitamin C, and polyphenolic antioxidants within the fruit. This accelerated metabolic process is accompanied by strawberries softening, increased risk of decay, and accelerated quality deterioration (Jiang, Zhao, Yang, & Fan, 2023). As shows in Fig. 3b, from day 0 to day 4, the respiration rate of strawberries decreased rapidly. After day 4, it showed a slow upward trend. This phenomenon may be attributed to the fact that in the later stages of storage, strawberries transition from full ripeness to the initial phase of decay, causing their respiration rate to temporarily increase. In addition, during storage, the respiration rate of strawberries in the 3EU@Ag-MOF/PLA film treatment group decreased the slowest (*p* < 0.05).

#### 3.1.4 The total phenol and ascorbic acid content in strawberries

During the postharvest oxidative browning process in strawberry, the consumption of polyphenols as substrates for polyphenol oxidase leads to a decrease in total phenol content (TPC) (Gul et al., 2024). The TPC of strawberries in all groups during storage showed a tendency of increasing and then decreasing (Fig. 3c). The TPC of strawberries in the control (8.38 ± 0.01 mg GAE/g), PLA film (8.49 ± 0.06 mg GAE/g), 1EU@Ag-MOF/PLA film (8.72 ± 0.05 mg GAE/g), 3EU@Ag-MOF/PLA film (8.89 ± 0.09 mg GAE/g) and 5EU@Ag-MOF/PLA film (8.75 ± 0.08 mg GAE/g) reached the maximum value on day 4 (*p* < 0.05). The increase could be explained by the fact that PLA film and EU@Ag-MOF/PLA films can limit the exposure of the fruit to oxygen, retaining compounds such as phenols (Wang et al., 2023). More intriguingly, the TPC of strawberries treated with the 3EU@Ag-MOF/PLA film was consistently higher than that of the other groups (*p* < 0.05). Research suggested that aromatic compounds in essential oils could bind to the active sites of PPO and PAL, thereby inhibited their enzymatic activity. This mechanism may helped increase the total phenolic content of fruits (Min et al., 2024). Meanwhile, the presence of Ag in the packaging protected strawberries from UV damage, reduced the production of ROS, and slowed down the oxidation process of strawberries.

The ascorbic acid (AsA) content degradation is facilitated by the presence of oxygen. Fig. 3d shows the trend of AsA content changes in strawberries during storage. The AsA content of each group strawberries gradually decreased from day 0 to day 10. Finally, AsA content in the control group, PLA film, 1EU@Ag-MOF/PLA film, 3EU@Ag-MOF/PLA film, and 5EU@Ag-MOF/PLA film decreased by 42.7 %, 41.0 %, 38.4 %, 30.4 %, and 38.1 %, respectively. These results suggested that the EU@Ag-MOF/PLA film acted as a barrier to oxygen, which reduced the rate of transpiration and consequently decreased the oxidation of ascorbic acid (Qin et al., 2024). Furthermore, EU@Ag-MOF contains the antioxidant component EU, which can provide a relatively antioxidant microenvironment for strawberries. It effectively inhibits the oxidation of AsA, allowing the AsA within the strawberries to be preserved. The higher the content of TPC and AsA in strawberries, the better their nutritional, antioxidant, color and health care value (Sinha, Gill, Jawandha, Kaur, & Grewal, 2021). The results showed that the 3EU@Ag-MOF/PLA film could most effectively preserve phenolic substances in strawberries during storage, while inhibited the oxidative loss of AsA (*p* < 0.05).

#### 3.1.5 The content of H_2_O_2_ and •O_2_- in strawberries

**Fig. 4:**
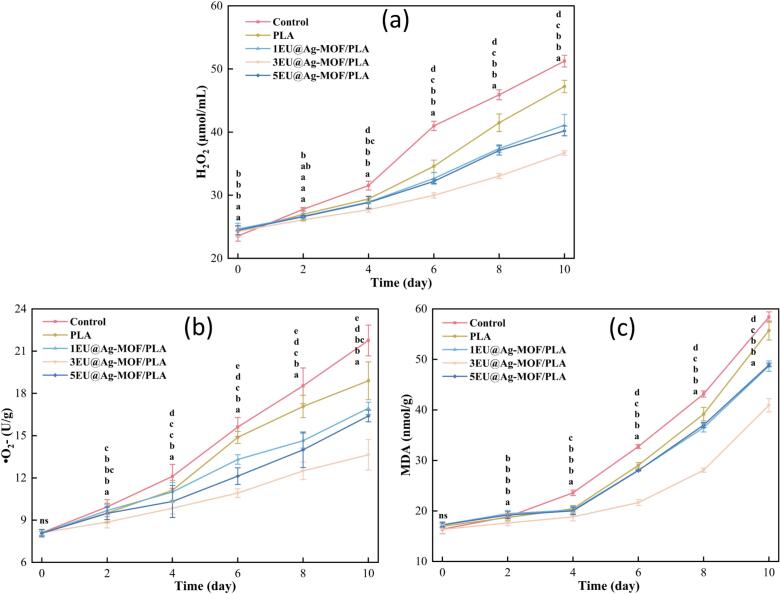
H_2_O_2_ (a), •O_2_- (b) and MDA (c) contents during strawberry storage. Each point represents the mean value, with error bars indicating the standard error of the mean. Statistical significance between different treatments at each time point is denoted by different letters, with a significance level set at *p* < 0.05. The arrangement of letters from top to bottom indicates the statistical ranking from highest to lowest value at each storage time. The notation “ns” indicates non-significant.

H₂O₂ and •O_2_- are two main oxygen species (ROS) radicals produced by fruits and vegetables during storage, which are both by-products of cellular metabolism and important signaling molecules (Sies & Jones, 2020). Excessive accumulation of ROS can disrupt the oxidative balance, leading to cell lysis and thereby causing damage to strawberry cells. In the first four days, both H₂O₂ (Fig. 4a) and •O_2_- (Fig. 4b) of strawberries showed a slowly upward trend. This was mainly due to the decreased respiration of strawberries during this period, which led to a reduction in the production of ROS. At the same time, ROS were partially removed by antioxidant enzymes. After the fourth day, the upward trend accelerated. The large accumulation of H₂O₂ and •O_2_- was attributed to the enhanced respiration of strawberries, which led to an intensified attack of ROS on cellular components and a weakening of the antioxidant system at this stage. The ROS content in EU@Ag-MOF/PLA groups was lower than that in the control (*p* < 0.05). Which may have been attributed to the presence of the EU. Tian et al. reported that tea tree essential oil regulated the fresh-cut pineapple physiological metabolism and activated antioxidant enzymes to break down the generated ROS radicals (Tian et al., 2024). Ali et al. decreased the H₂O₂ and •O_2_- accumulation by combining gum arabic and γ-aminobutyric acid in Kinnow (Ali et al., 2023).

#### 3.1.6 MDA content in strawberries

Fig. 4c shows the trend of strawberries malondialdehyde (MDA) content during storage. In the early stages of strawberry ripening, mild lipid peroxidation occurred, accompanied by the production of small amounts of MDA. The MDA levels in all groups showed a gradual increase. By the fourth day, the rate of MDA accumulation accelerated. This phenomenon occurred because fully ripened strawberries began to decay, leading to the gradual breakdown of cellular membrane systems. As a result, reactive ROS attack increased, lipid oxidation deepened, and MDA production accelerated (Chen et al., 2025).

Compared with day 0, MDA contents in the control, PLA film, 1EU@Ag-MOF/PLA film, 3EU@Ag-MOF/PLA film and 5EU@Ag-MOF/PLA film increased by 43.9 %, 20 %, 16.8 %, 14.6 %, and 15.6 % on day 4. From day 4 to day 10, the MDA contents in strawberries increased by 147 %, 173 %, 141 %, 118 %, and 145 %, respectively. The accumulation of MDA in strawberries increased cell membrane permeability, leading to the leakage of cellular contents. This damaged strawberry tissues and accelerated quality deterioration. The MDA content analysis across groups demonstrated that EU@Ag-MOF/PLA significantly enhanced antioxidant effects, effectively inhibited MDA production in strawberries and reduced lipid peroxidation (*p* < 0.05). Consequently, this delayed the quality degradation of strawberries, with the 3EU@Ag-MOF/PLA film showing particularly notable effectiveness (*p* < 0.05).

#### 3.1.7 POD, CAT, SOD and PPO in strawberries

**Fig. 5:**
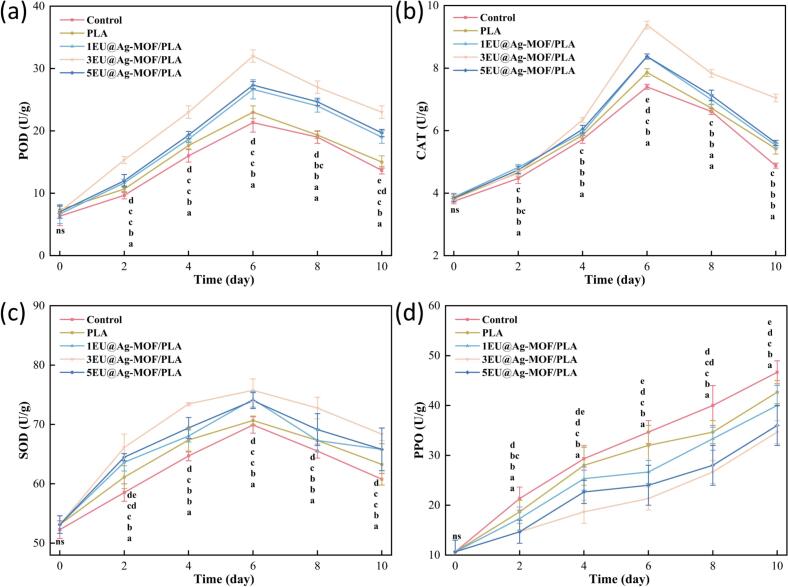
POD (a), CAT (b), SOD (c) and PPO (d) contents during strawberry storage. Each point represents the mean value, with error bars indicating the standard error of the mean. Statistical significance between different treatments at each time point is denoted by different letters, with a significance level set at *p* < 0.05. The arrangement of letters from top to bottom indicates the statistical ranking from highest to lowest value at each storage time. The notation “ns” indicates non-significant.

SOD, POD, and CAT are antioxidant enzyme systems in strawberry fruits, which are associated with scavenging ROS and mitigating oxidative damage in strawberries from ripening to spoilage. To avoid being harmed by reactive oxygen species, strawberries have corresponding defense enzyme systems such as POD, SOD and CAT, together with total phenols, ascorbic acid and other antioxidant substances, jointly play a role in eliminating ROS (Duan, Li, Meng, Wang, & Wu, 2021). From Fig. 5a, b and c, the activities of POD, CAT and SOD of strawberries in each group showed an upward trend until day 6. Which was mainly due to the fact that in the early stage of strawberry ripening, the strawberry produced POD, SOD and CAT in response to eliminate the generated ROS, in order to maintain the cellular homeostasis. After that, POD, SOD and CAT showed a downward trend. This resulted mainly due to the collapse of enzyme system of the strawberries, resulting in ROS accumulation and accelerated strawberry cell damage (Obianom Dharini, 2019; Shinga, Fawole, & Pareek, 2025). Compared with the control and PLA groups, EU@Ag-MOF/PLA groups were able to keep the antioxidant enzymes of strawberries at higher levels during storage (*p* < 0.05). The antioxidant component EU in EU@Ag-MOF helped maintain a relatively balanced redox environment within strawberries. This stable condition initially promoted a gradual increase in the activities of POD, SOD, and CAT enzymes, reaching a higher peak level. Due to the sustained release of EU providing prolonged antioxidant protection, the decline in POD, SOD, and CAT activities occurred at a slower rate during later stages (*p* < 0.05).

PPO is a strawberry oxidation-related enzyme that is mainly involved in catalyzing the oxidation of phenolics in strawberry fruits, and the quinones produced by this process can lead to browning and quality deterioration of strawberries (Chi et al., 2024). From Fig. 5d, the PPO values of strawberries in all groups during storage showed an increasing trend. The increased PPO activity was caused by the exposure of phenolic substrates and slight cell damage in the strawberries from incomplete ripening to full ripening. In addition, from ripening to spoilage, the PPO activity was further enhanced due to the rupture of strawberry cell membranes releasing phenolic substances or the infection of pathogenic microorganisms (Qin et al., 2025). It can be seen that the PPO activity of strawberries treated with EU@Ag-MOF/PLA films always remained at a lower level during the storage period compared with the control and PLA groups (*p* < 0.05). The EU@Ag-MOF/PLA packaging demonstrated superior barrier properties, effectively slowed down the ingress of oxygen and moisture, thereby inhibited PPO activity. Furthermore, the 3EU@Ag-MOF/PLA film exhibited the strongest antioxidant performance, corresponded to the slowest (*p* < 0.05) rise in PPO levels (Qin et al., 2024).

### 3.2 Effect of 3EU@Ag-MOF/PLA film on postharvest ROS metabolism of inoculated strawberries

#### 3.2.1 The changes of H_2_O_2_, •O_2_- and MDA contents in inoculated strawberries

**Fig. 6:**
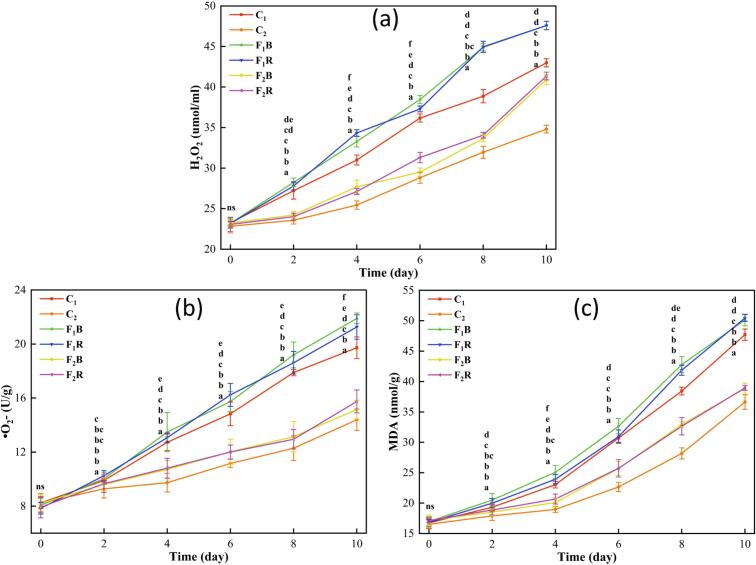
H_2_O_2_ (a), •O_2_- (b) and MDA (c) contents of strawberries in groups. C_1_, C_2_, F_1_R, F_1_B, F_2_R and F_2_B. C_1_ was the uninoculated strawberries packaged with PLA film, C_2_ was the uninoculated strawberries packaged with 3EU@Ag-MOF/PLA film, F_1_R was the inoculated with *R. stolonifera* strawberries packaged with PLA film, F_2_R was the inoculated with *R. stolonifera* strawberries packaged with 3EU@Ag-MOF/PLA film, F_1_B was the inoculated with *B. cinerea* strawberries packaged with PLA film, F_2_B was the inoculated with *B. cinerea* strawberries packaged with 3EU@Ag-MOF/PLA film. Each point represents the mean value, with error bars indicating the standard error of the mean. Statistical significance between different treatments at each time point is denoted by different letters, with a significance level set at *p* < 0.05. The arrangement of letters from top to bottom indicates the statistical ranking from highest to lowest value at each storage time. The notation “ns” indicates non-significant.

H₂O₂ and •O_2_- are key components of ROS, primarily generated by enzymes such as NADPH oxidases located on strawberry cell membranes. Excessive ROS can attack unsaturated fatty acids in the membrane, triggering lipid peroxidation and subsequently producing MDA (Sun et al., 2023). The levels of H₂O₂ (Fig. 6a), O₂^•-^ (Fig. 6b), and MDA (Fig. 6c) in all groups exhibited a gradual increase during storage. C_1_ was the uninoculated strawberries packaged with PLA film, C_2_ was the uninoculated strawberries packaged with 3EU@Ag-MOF/PLA film, F_1_R was the inoculated with *R. stolonifera* strawberries packaged with PLA film, F_2_R was the inoculated with *R. stolonifera* strawberries packaged with 3EU@Ag-MOF/PLA film, F_1_B was the inoculated with *B. cinerea* strawberries packaged with PLA film, F_2_B was the inoculated with *B. cinerea* strawberries packaged with 3EU@Ag-MOF/PLA film. Among them, the C_2_ group showed the lowest concentrations, while the F_2_R and F_2_B groups had higher levels than C_2_ (*p* < 0.05). Similarly, the F_1_R and F_1_B groups displayed elevated concentrations compared to the C_1_ group. This phenomenon primarily occurred because strawberries inoculated with pathogenic fungi tended to activate oxidative stress mechanisms to combat pathogen invasion, leading to a surge in H₂O₂ and O₂^•-^. The accumulation of these reactive oxygen species subsequently triggered an increase in MDA content. Furthermore, strawberries treated with the 3EU@Ag-MOF/PLA film (F_2_R and F_2_B groups) demonstrated lower H₂O₂, O₂^•-^, and MDA levels compared to those treated with PLA (*p* < 0.05). This suggested that the 3EU@Ag-MOF/PLA film effectively suppressed the growth of *B. cinerea* and *R. stolonifera* (*p* < 0.05). The observed effect may have been attributed to the sustained release of EU from Eu@Ag-MOF nanoparticles within the film, which inhibited the production of H₂O₂, O₂•^−^, and MDA (Qin et al., 2025).

#### 3.2.2 The changes of POD, SOD, CAT and PPO in inoculated strawberries

**Fig. 7:**
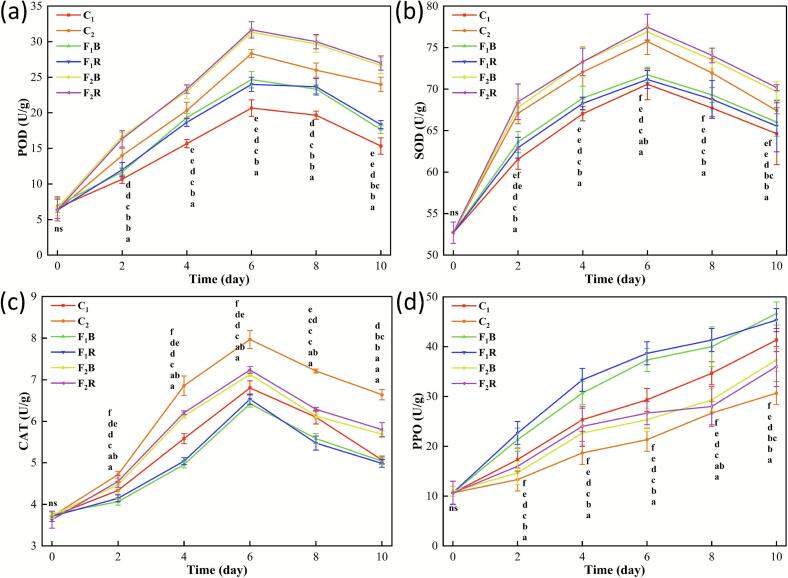
POD (a), SOD (b), CAT (c) and PPO (d) contents of strawberries in groups. C_1_, C_2_, F_1_R, F_1_B, F_2_R and F_2_B. C_1_ was the uninoculated strawberries packaged with PLA film, C_2_ was the uninoculated strawberries packaged with 3EU@Ag-MOF/PLA film, F_1_R was the inoculated with *R. stolonifera* strawberries packaged with PLA film, F_2_R was the inoculated with *R. stolonifera* strawberries packaged with 3EU@Ag-MOF/PLA film, F_1_B was the inoculated with *B. cinerea* strawberries packaged with PLA film, F_2_B was the inoculated with *B. cinerea* strawberries packaged with 3EU@Ag-MOF/PLA film. Each point represents the mean value, with error bars indicating the standard error of the mean. Statistical significance between different treatments at each time point is denoted by different letters, with a significance level set at *p* < 0.05. The arrangement of letters from top to bottom indicates the statistical ranking from highest to lowest value at each storage time. The notation “ns” indicates non-significant.

In strawberries, H₂O₂ and •O_2_- act as signaling molecules to stimulate the upregulation of the activities of three antioxidant enzymes, namely POD, SOD, and CAT. Through a synergistic effect, they eliminate excessive ROS, maintain the redox balance, and protect strawberry cells from oxidative damage (Lu et al., 2025; Meng et al., 2024). Although PPO indirectly affects oxidative homeostasis by oxidizing phenolic substrates, but the quinones produced in this process trigger strawberry browning (Tilley, McHenry, McHenry, Solah, & Bayliss, 2023). The POD (Fig. 7a), SOD (Fig. 7b) and CAT (Fig. 7c) activities of inoculated strawberries in all groups showed an increasing initially and then decreasing. And reached the maximum value on day 6. Both POD and SOD activities of strawberries in group C_1_ were the lowest (*p* < 0.05). Compared to the C_1_ group, on day 6, the POD activity in strawberries from the F_1_R and F_1_B groups decreased by 16.1 ± 0.75 % and 19.4 ± 1.01 %, respectively. While the SOD activity showed reductions of 0.8 ± 0.02 % and 1.6 ± 0.07 %, respectively. Additionally, the POD and SOD enzyme activities in strawberries from the F_2_R and F_2_B groups were higher than those in the C_2_ group (*p* < 0.05). By day 6, the POD activity in F_2_R and F_2_B strawberries had increased to 31.67 ± 1.15 U/g and 31.34 ± 0.58 U/g, respectively, while the SOD activity reached 77.47 ± 1.56 U/g and 76.90 ± 0.92 U/g (*p* < 0.05). Meanwhile, the activities of POD and SOD in group C_2_ were only 28.34 ± 0.57 U/g and 75.75 ± 1.86 U/g respectively. This phenomenon occurred because infection by *B. cinerea* and *R. stolonifera* triggered the activation of POD and SOD enzymes in strawberries. These enzymes helped eliminate the excessive accumulation of ROS caused by pathogen infection, thereby delayed oxidative damage in the fruit. The 3EU@Ag-MOF/PLA film effectively maintained strawberry POD and SOD activities at elevated levels to mitigate spoilage, particularly following pathogenic infection (*p* < 0.05).

As shows in Fig. 7c, the CAT activity in strawberries from the C_1_ group was higher than that in the F_1_R and F_1_B groups. On day 6, the CAT activity in C_1_ strawberries exceeded that of F_1_R and F_1_B by 0.27 U/g and 0.38 U/g, respectively. Additionally, the CAT activity in F_2_R and F_2_B groups was lower than in the C_2_ group (*p* < 0.05). By day 6, the CAT activity values for C_2_, F_2_R, and F_2_B strawberries were 7.97 U/g, 7.24 U/g, and 7.13 U/g, respectively. As another key antioxidant enzyme in strawberries, the CAT activity in C_1_ and C_2_ groups surpassed that of F_1_R, F_1_B, F_2_R, and F_2_B groups, exhibited a trend opposite to that of POD and SOD (*p* < 0.05). This opposite trend in CAT activity may reflect the accumulation of H₂O₂ as a defensive signaling molecule during pathogen infection (Najafi, Esfahani, Vatandoost, Hassanzadeh-Khankahdani, & Moeini, 2024). The temporary suppression of CAT could help sustain H₂O₂-mediated defense responses, although further evidence would be needed to confirm this mechanism. Similar reported that under the infection of black smut in sugarcane, the CAT activity of both susceptible and resistant sugarcane genotypes decreased (Peters et al., 2017). In addition, infection by *B. cinerea* and *R. stolonifera* triggers the secretion of effector proteins by these pathogens, which inhibit strawberry CAT activity, thereby weakening oxidative defense and facilitating pathogen invasion. The CAT activity assay demonstrated that the 3EU@Ag-MOF/PLA film effectively maintained higher CAT activity in pathogen-infected strawberries, contributed to the preservation of their oxidative defense capacity (*p* < 0.05).

Fig. 7d reveals a gradual increase in PPO activity across all groups. The C_1_ group exhibited the lowest PPO activity (*p* < 0.05). The C_2_ group surpassed both F_2_R and F_2_B, whereas F_1_R and F_1_B demonstrated even greater activity than C_2_ (*p* < 0.05). This phenomenon occurred because pathogen infection triggered the release of phenolic compounds from strawberry cell membranes, which directly interacted with PPO, thereby enhanced enzymatic reactions and elevated PPO activity. Additionally, pathogen infection accelerated strawberry ripening and decay, further increased PPO activity (Zhang et al., 2023). Experimental results demonstrated that the 3EU@Ag-MOF/PLA film effectively suppressed the PPO activity, delayed browning, and exhibited protective effects for pathogen-infected strawberries (*p* < 0.05).

### 3.3 Effect of 3EU@Ag-MOF/PLA film on microbial community composition of inoculated strawberries

#### 3.3.1 Alpha diversity analysis

Alpha diversity analysis serves as a crucial method in microbiome research, specifically designed to evaluate species diversity within a single microbial community (Alqahtani et al., 2024). The Alpha diversity indices of bacterial and fungal communities in strawberries from groups C_1_, C_2_, F_1_R, F_2_R, F_1_B, and F_2_B after 10 days are presented in Table S1-S4. The tables showed that the Goods Coverage values for both bacteria and fungi in all groups reached 1, indicating that the sequencing depth sufficiently captured all microbial species present in the samples. After 10 days of storage, the Observed Species counts for bacterial communities were 14 (C_1_), 13 (C_2_), 67 (F_1_R), 38 (F_2_R), 63 (F_1_B), and 17 (F_2_B), while the fungal counts were 60 (C_1_), 23 (C_2_), 9 (F_1_R), 7 (F_2_R), 57 (F_1_B), and 8 (F_2_B). These results demonstrated an increase in OTUs for strawberries inoculated with the pathogenic fungi *B. cinerea* and *R. stolonifera*. Notably, bacterial OTU counts in the F_2_B and F_2_R groups were lower than those in F_1_B and F_1_R (*p* < 0.05). Conversely, fungal OTU numbers in F_1_B, F_1_R, F_2_B, and F_2_R were reduced compared to the control groups (C_1_ and C_2_). Consistent with bacterial trends, fungal OTUs in F_2_B and F_2_R were also fewer than in F_1_B and F_1_R. The bacterial species richness (Chao 1 = 41.5 and 23) in strawberries from the F_2_R and F_2_B groups was lower than that in the F_1_B and F_1_R groups (Chao 1 = 68.5 and 65) (*p* < 0.05). The bacterial diversity index (Shannon = 1.494) in the F_2_R group was higher than that in the F_1_B group (Shannon = 1.469), while the F_2_B group showed a lower diversity index (Shannon = 1.012) compared to the F_1_R group (Shannon = 1.352) (*p* < 0.05). Similarly, the bacterial evenness (Simpson = 0.539) in the F_2_R group was higher than in the F_1_B group (Simpson = 0.472), whereas the F_2_B group exhibited lower evenness (Simpson = 0.412) than the F_1_R group (Simpson = 0.493) (*p* < 0.05). For fungal communities, the species richness, diversity index, and evenness were consistently higher in the F_1_R and F_1_B groups than in the F_2_R and F_2_B groups (*p* < 0.05).

The analysis of microbial community diversity provides several important insights into the antimicrobial of 3EU@Ag-MOF/PLA film in preserving strawberry quality. First, the composite film demonstrated broad-spectrum antimicrobial activity, effectively suppressing both bacterial and fungal proliferation during refrigerated storage. This was evidenced by the consistently lower microbial richness in 3EU@Ag-MOF/PLA-packaged samples compared to their PLA-packaged, regardless of pathogen inoculation status (*p* < 0.05). Second, the composite film exhibited particularly potent antifungal activity. This pronounced effect on fungal communities explains the previously observed improvements in quality parameters and reduced oxidative stress in 3EU@Ag-MOF/PLA-packaged strawberries, as fungal pathogens represent primary drivers of postharvest deterioration. Third, the temporal dynamics of microbial diversity metrics reveal that the 3EU@Ag-MOF/PLA film not only reduced absolute microbial abundance but also modulated community succession patterns during storage. The maintenance of more stable diversity indices in 3EU@Ag-MOF/PLA-packaged samples suggests that the composite film helps preserve ecological balance in the fruit microbiome, potentially allowing beneficial or commensal microorganisms to persist while suppressing pathogenic species.

#### 3.3.2 Analysis of species composition of bacterial groups

**Fig. 8:**
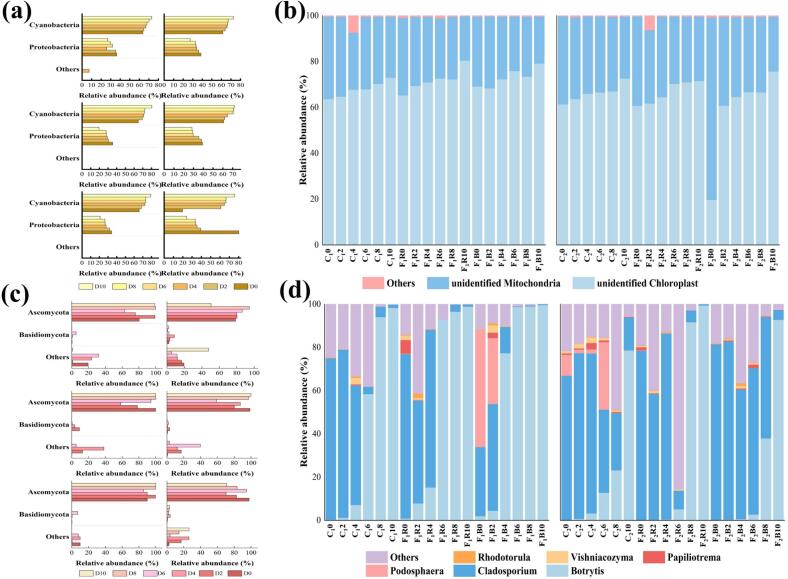
Relative abundance of strawberry bacterial phylum levels (a), bacterial genus levels (b), fungal phylum levels (c) and fungal genus levels (d) in the C_1_, C_2_, F_1_R, F_2_R, F_1_B, and F_2_B groups during storage. C_1_ was the uninoculated strawberries packaged with PLA film, C_2_ was the uninoculated strawberries packaged with 3EU@Ag-MOF/PLA film, F_1_R was the inoculated with *R. stolonifera* strawberries packaged with PLA film, F_2_R was the inoculated with *R. stolonifera* strawberries packaged with 3EU@Ag-MOF/PLA film, F_1_B was the inoculated with *B. cinerea* strawberries packaged with PLA film, F_2_B was the inoculated with *B. cinerea* strawberries packaged with 3EU@Ag-MOF/PLA film.

Fig. 8a shows the relative abundance of strawberry bacteria based on phylum level during storage. The dominant phyla of strawberry bacteria in all groups were mainly *Cyanobacteria* and *Proteobacteria*, which are prevalent in habitats such as soil, plants, water, and the atmosphere (Nie et al., 2023). The relative abundance of *Cyanobacteria* in all groups showed a gradual upward trend, accounted for 63.72–73.02 % (C_1_), 61.42–72.72 % (C_2_), 64.69–80.45 % (F_1_R), 60.78–71.60 % (F_2_R), 65.84–79.21 % (F_1_B) and 19.72–75.73 % (F_2_B), respectively. With the exception of group C_1_, other groups exhibited a gradual decline in the relative abundance of *Proteobacteria* in strawberries. The relative abundance ranges of *Proteobacteria* were: 25.72–36.28 % for C_1_, 27.28–38.57 % for C_2_, 19.55–35.31 % for F_1_R, 28.40–39.22 % for F_2_R, 20.79–34.14 % for F_1_B, and 24.26–80.19 % for F_2_B. Overall, the proportions of *Cyanobacteria* and *Proteobacteria* in the total sequences were lower in the F_2_R and F_2_B strawberry groups compared to the F_1_R and F_1_B groups (*p* < 0.05). These results indicated that, even after pathogen inoculation, the 3EU@Ag-MOF/PLA membrane effectively suppressed the relative abundance of *Cyanobacteria* and *Proteobacteria* in strawberries.

Fig. 8b shows the relative abundance of bacteria based on genus level in strawberries within 10 days. The predominance of unidentified *Chloroplast* and unidentified *Mitochondria* sequences across all samples likely represented strawberries chloroplast and mitochondrial DNA. The consistent presence of these sequences throughout storage indicated the persistence of strawberries cellular material. The distribution of bacterial species remained relatively low throughout the storage process, further confirmed that fungal infection had the greatest impact on strawberries.

#### 3.3.3 Species composition analysis of fungal groups

As shows in Fig. 8c, the relative abundance of strawberry fungi in groups based on the phylum level was detected, in which *Ascomycota* and *Basidiomycota* were the main dominant phyla. The proportion of *Ascomycota* and *Basidiomycota* in the total sequences was 50.44–99.99 % and 0–9.13 %, respectively. The relative abundance of *Ascomycota* in groups C_1_, F_1_R and F_1_B was higher than that in groups C_2_, F_2_R and F_2_B (*p* < 0.05). The results indicated that 3EU@Ag-MOF/PLA film had a certain inhibitory effect on the relative abundance of the dominant fungal phyla in strawberries.

Fig. 8d shows the relative abundance of strawberry fungi based on genus level in groups. Six major dominant fungi were obtained, including the genera of *Botrytis* (0.13–99.66 %), *Cladosporium* (0.02–86.03 %)*, Podosphaera* (0–54.23 %)*, Papiliotrema* (0–6.21 %)*, Vishniacozyma* (0–3.40 %) and *Rhodotorula* (0–1.97 %). For all groups, *Botrytis* and *Cladosporium* had the highest percentage during storage. Gray mold caused by *Botrytis cinerea* is one of the most important economic diseases in strawberry production, which causing fruit rot and reducing yields (Priyadarshi, Jayakumar, de Souza, Rhim, & Kim, 2024). *Cladosporium* is a saprophytic fungus that is able to utilize the organic matter on the surface of strawberry for growth and reproduction (Prasannath, Shivas, Galea, & Akinsanmi, 2021). The relative abundance of *Botrytis* in strawberries of all groups showed a gradual increase, and the values of C_1_, F_1_R and F_1_B groups were notable increase than the C_2_, F_2_R and F_2_B groups (Fig. 8d). The strawberry pathogen *B. cinerea* belongs to the *Botrytis* genus. As a result, the 3EU@Ag-MOF/PLA film demonstrated efficacy in suppressing the relative abundance of *Botrytis*. Similar study has shown that the coating composed of sodium benzoate and chitosan could inhibited the reproduction of the main pathogenic fungi (*Alternaria* and *Cladosporium*) of jujube fruit, and effectively reduced their relative abundance (Liu et al., 2025).

## 4 Conclusion

This study demonstrated that 3EU@Ag-MOF/PLA film offers a promising approach to enhancing the quality of postharvest strawberries. Firstly, 3EU@Ag-MOF/PLA film effectively reduces the respiration rate of strawberries, thereby inhibiting over-ripening and softening of the fruit. Secondly, maintains the activities of antioxidant enzymes (POD, CAT, and SOD) in strawberries at a high level, while suppressing the activity of PPO. Meanwhile, increases the levels of secondary metabolites (AsA and total phenols). These changes help mitigate the cytotoxic effects of reactive oxygen species and enhance cellular protection. Thirdly, 3EU@Ag-MOF/PLA film extends the shelf life of strawberries by effectively reduce pathogen growth and microbial species composition. Moreover, 3EU@Ag-MOF/PLA film is expected to reduce white pollution caused by traditional plastics from the source. Future research should explore the scalability and cost-effectiveness of 3EU@Ag-MOF/PLA film production, and investigate consumer acceptance.

## CRediT authorship contribution statement

**Yuyue Qin:** Writing – review & editing, Validation, Supervision, Resources, Project administration, Funding acquisition, Conceptualization. **Yurou Wang:** Writing – original draft, Methodology, Data curation. **Yiwei Huang:** Methodology, Investigation, Data curation. **Haiyan Chen:** Supervision, Project administration. **Yongliang Zhuang:** Software. **Qiuming Liu:** Supervision, Conceptualization. **Thanapop Soteyome:** Writing – review & editing, Resources, Conceptualization. **Bifen Zhu:** Writing – review & editing, Visualization, Supervision, Resources, Conceptualization. **Charles Brennan:** Writing – review & editing, Resources.

## Funding declaration

The authors thank the support from the Yunnan International Joint Laboratory Foundation of Green Food Processing, China (No. 202403AP140044), Yunnan Province International Science and Technology Special Correspondent Foundation of China (No. 202403AK140001), and 10.13039/501100001809National Natural Science Foundation of China (No.22268024).

## Declaration of competing interest

The authors declare that there is no conflict of interest.

## Data Availability

Data are contained within the article.
